# Understanding and attenuating pandemic-related disruptions: a plan to reduce inequalities in child development

**DOI:** 10.17269/s41997-021-00584-7

**Published:** 2022-01-28

**Authors:** Sylvana M. Côté, Marie-Claude Geoffroy, Catherine Haeck, Isabelle Ouellet-Morin, Simon Larose, Nicholas Chadi, Kate Zinszer, Lise Gauvin, Benoit Mâsse

**Affiliations:** 1 School of Public Health, Université de Montréal, Montréal, Québec Canada; 2 CHU Sainte-Justine, Montréal, Québec Canada; 3 Department of Educational and Counselling Psychology, McGill University, Montréal, Québec Canada; 4 Douglas Hospital Research Centre, Montréal, Québec Canada; 5 Department of Economics, University of Québec in Montréal, Montréal, Québec Canada; 6 School of Criminology, University of Montreal, Montréal, Québec Canada; 7 Département d’études sur l’enseignement et l’apprentissage, Université Laval, Québec, Québec Canada; 8 Department of Pediatrics, University of Montreal, Montréal, Québec Canada; 9 Centre de recherche du centre hospitalier de l’Université de Montréal, Montréal, Québec Canada

**Keywords:** Children, Education, COVID-19, Mental health, Prevention, Evaluation, Enfants, éducation, COVID-19, santé mentale, prévention, évaluation

## Abstract

The Secretary General of the United Nations described the impact of COVID-19-related school closures as a “generational catastrophe.” What will be the legacy of the 2020–2021 pandemic-related disruptions in 5, 10, 20 years from now, as regards education and well-being of children and youth? Addressing the disproportionate impact on those growing up in socio-economically disadvantaged areas or on those with pre-existing learning challenges is key to sustainable recovery. This commentary builds on the four literature reviews presented in this *Special Section on a Pandemic Recovery Plan for Children* and proposes strategies to understand and attenuate the impact of pandemic-related lockdown measures. Importantly, we need a monitoring strategy to assess indicators of child development in three areas of functioning: education and learning, health, and well-being (or mental health). Surveillance needs to begin in the critical prenatal period (with prenatal care to expectant parents), and extend to the end of formal high school/college education. Based on child development indicators, a stepped strategy for intervention, ranging from all-encompassing population-based health and education promotion initiatives to targeted prevention programs and targeted remedial/therapeutic interventions, can be offered. As proposed in the UN plan for recovery, ensuring healthy present and future generations involves a concerted and intensive intersectoral effort from the education, health, psychosocial services, and scientific communities.

## Résumé

Selon les Nations Unis, les perturbations scolaires associées à la pandémie de COVID-19 pourraient mener à une « catastrophe générationnelle » en gaspillant un potentiel humain incalculable, sapant des décennies de progrès et exacerbant des inégalités bien ancrées. Le déploiement du vaccin chez les moins de 12 ans n’a pas débuté avant la rentrée scolaire 2021, ce qui laisse présager de nombreuses perturbations pour cette 3e année scolaire pandémique. Quel sera le legs des perturbations entrainées par la pandémie de COVID-19 en matière d’éducation et de bien-être dans 5, 10, ou 20 ans? Une relance durable dépendra des mesures prises pour prévenir l’impact négatif disproportionné sur les enfants/adolescents de milieux socioéconomiques défavorisés et ceux qui ont des défis d’apprentissage préexistants. Ce commentaire propose des stratégies pour comprendre et atténuer l’impact des perturbations pandémiques en se fondant sur les quatre articles de la présente « *Section spéciale sur un plan de relance pour les enfants suite à la pandémie »*. Trois lignes d’action prioritaires émergent à la lumière des connaissances scientifiques actuelles. Premièrement, le contexte pandémique met en évidence la nécessité d’accéder à données intersectorielles (éducation, santé, services psychosociaux) permettant de distinguer les conséquences à court et à long terme. Deuxièmement, il faut être prêt à déployer une stratégie interventionnelle par étape, avec des interventions universelles en promotion, jusqu’aux interventions plus ciblées et intensives. Troisièmement, il faudra mettre en place des programmes particuliers pour les enfants/adolescents de milieux défavorisés et pour ceux qui présentent des facteurs de risque personnels (défis de santé mentale, retards d’apprentissage). À titre d’exemple, le tutorat scolaire devrait être facilement accessible dans tous les milieux défavorisés. Tel que proposé dans le plan de relance de l’ONU, un effort concerté, intensif et intersectoriel de la part des sciences de l’éducation, de la santé, et des services psychosociaux sera nécessaire pour assurer la santé et l’éducation des générations présentes et futures.

The Secretary General of the United Nations described the impact of COVID-19-related school closures as a “generational catastrophe” (United Nations, 2021a). What will be the legacy of the 2020–2021 pandemic in 5, 10, 20 years from now, as regards education and well-being? Will children, teens, and young people recuperate quickly, without specific intervention? Or will they suffer long-term consequences, requiring intensive support? Will societal expectations be reduced, such that the negative impact on education will not be strikingly evident? This *Special Section on a Pandemic Recovery Plan for Children* presents four review papers on the consequences of the pandemic for children in terms of education (learning loss), healthy lifestyles, mental health and well-being, and the potential of early preventive strategies. Based on these reviews, this commentary summarizes the area of concerns for children and youth, and proposes a monitoring system to understand the short- and long-term impacts, as well as a series of interventions to improve the health and education in the general population and to catch those with delays.

As outlined in the four reviews in this special section, surveillance of child development in terms of education and well-being will be key throughout the 2020s and 2030s. Newly minted international data tend to confirm what many expected—a disproportionate impact on certain populations depending on specific vulnerabilities and areas of concern. The size of the impact is likely to vary according to the intensity of the pandemic in any given geographical region. We need to fill in key data gaps before we can grasp the full magnitude of the effect. With the literature reviews presented, this special section summarizes childhood developmental dimensions on which to focus recovery efforts and outlines four levels of intervention to attenuate the impact of the pandemic, ranging from universal health and education promotion to specific therapeutic intervention.

## Defining priorities: who and what should be the focus of recovery efforts

Virtually all children and adolescents have experienced major disruptions in schooling and in social and physical activities and all ecological spheres of human functioning have been affected. From the reviews emerges a nuanced picture of the impact of pandemic-related disruptions on learning losses, mental health/well-being, and healthy lifestyles.

### Concern 1: Learning loss

For children and youth, the COVID-19 pandemic has not, thus far, presented major concerns in terms of physical health. But the loss of time in a structured learning environment is particularly concerning for children at risk because of disadvantaged socio-economic status or special needs. Children in families with low education level are least likely to receive support from family or private services.

As of March 29, 2021, schools in Canada had closed for 40 weeks (13 weeks full-time and 27 weeks part-time for high school students); furthermore, there were regular class closures due to outbreaks (Gallagher-Mackay et al., 2021). Online teaching was deployed unevenly and engaged less than 50% of students, as compared with 80% in class (Naitre et Grandir, 2021). Haeck and Larose (2021) expect significant learning losses in all children, with a performance gap between children with and without academic problems increasing by 30%. The few good-quality studies indeed highlight a decrease in school grades ranging from 2 to 6 percentiles for all children and from 4.5 to 17 percentiles for children from families where parents have a low level of education or among those attending schools from disadvantaged backgrounds (Lewis et al., 2021; Engzell et al., 2021).

Importantly, preliminary evidence suggests that graduation standards have been adjusted to match general levels of population performance. For example, high school graduation rates in various countries were no different or even higher in 2020 than in previous years (UNESCO et al., 2020). In addition, school curricula have been adjusted to focus on essential learning. Thus, the consequences of the pandemic may not be felt unless standardized tests compare the state of learning pre- and post-pandemic. These assessments will help monitor the medium-term effects of school disruptions as well as progress in remediation, particularly as concerns the most vulnerable students.

### Concern 2: Mental health and well-being

The second area of concern is mental health and well-being. Here again, the impact of the pandemic is not uniform. A meta-analysis of 136 studies conducted during the first year of the pandemic (between March 2020 and February 2021) suggests that rates of clinically elevated depression and anxiety among youth (<18 years) have doubled compared to prepandemic levels. One in 4 youth globally are experiencing depression symptoms, while 1 in 5 youth are experiencing anxiety symptoms. These estimates, which increased over time, are higher for older children and for girls (Racine et al., 2021). Of note, the population most at risk may not necessarily be the one with the most severe mental health problems prior to the pandemic (Watkins-Martin et al., 2021). For instance, 48% of parents report de novo mental health challenges in their children (Inspiring Healthy Futures, 2021).

Similarly, patterns of substance use are not increasingly worse across the board, but rather depend on the type and pattern of use. Specifically, there are indications of decreases in binge drinking among young adults (Pocuca et al., under review). This probably relates to the lack of social opportunity for consuming alcohol, due to pandemic lockdowns. Data on suicide also vary, depending on a number of parameters such as type and source of data. Chadi et al. (2021) in this special section note an increase in suicide attempts (according to hospital data), but no increase in rates of death by suicide. Thus, research is needed to put together the pieces and understand who is impacted and, most importantly, to document the putative lasting effects of pandemic-related disruptions in services and daily activities on mental health. 

### Concern 3: Healthy lifestyle

Educational settings provide norms and rhythms essential to regulating social interactions and healthy behaviours such as eating and physical activity, during both school and extracurricular activities. As pointed out by Gauvin et al. (2021) in this section, pandemic-related school disruptions have led to marked reductions in physical activity, accompanied by increased sedentary behaviour and screen time, and increased food intake and unhealthy snacking. Deleterious effects in physical activity appear to be more pronounced in socio-economically vulnerable groups, particularly in urban areas (Gauvin et al., 2021). It is therefore not surprising that a recent American study (*n* = 191,509) reports that children 5–15 years of age gained on average 2.3 kg more than they did during a pre-pandemic comparison period, and that rates of obesity increased by 8.7% (Woolford et al., 2021).

There has been a strong global trend for increased screen time since the pandemic began (Sultana et al., 2021), in part because some or most schoolwork was remote and required online access. However, the concern with the use of electronic devices is not as much related to schoolwork as it is with leisure activities. Digital literacy should be among our top educational priorities (OECD, 2021). Digital literacy refers to individual interest, attitude, and ability in the use of digital technology and communication tools in order to appropriately access, manage, integrate, analyze, and evaluate information, construct new knowledge, and create and communicate with others (Government of British Columbia, 2021). In children, digital literacy should be part of educational curricula (OECD, 2021), while the use of electronic devices for play should be structured and supervised by adults.

## A unified monitoring strategy

As stated in the plan for post-pandemic recovery proposed by the UN, investment in data systems and infrastructures is going to be the linchpin in recovery efforts (United Nations, 2020). All authors of the review articles in this special section underscore the importance of research efforts to distinguish transient from persistent effects of the pandemic.

Deliberate and concerted efforts need to be deployed in order to overcome two important data gaps and challenges: (1) the lack of intersectoral data; and (2) the lack of longitudinal data. First, the necessary data are collected and stored in different database systems (research data; administrative databases in education, health, social services) by different parties (researchers and distinct ministries). The first challenge is encountered when a researcher testing a tutoring program for improvements in academic achievement and reduction of psychostimulant medications needs data from educational and medical sources, which are not matched. The second challenge (longitudinal data) arises whenever attempting to map the care or education trajectory of a child across developmental periods. For instance, to understand whether a change in early child care and education policy (i.e., daycare services) is related to better school readiness, higher graduation rates, and lower use of psychosocial services, one needs to access data on child care use in the first 5 years as well as health, education, and administrative data during the elementary school years. A monitoring system allowing to track indicators of learning and well-being longitudinally is needed.

In most territories/provinces, the lack of a unified longitudinal monitoring system is an important obstacle to the implementation of early preventive or therapeutic services. A notable exception is the *Manitoba Population Research Data Repository* (University of Manitoba, 2020). The Research Data Repository is a comprehensive collection of administrative, registry, survey, and other data primarily relating to residents of Manitoba. It was developed to describe and explain patterns of health care and profiles of health and illness, facilitating interdisciplinary research in areas such as health care, education, social services, and justice. A unique identifier—the Personal Health Identification Number (PHIN)—facilitates linkages from birth to old age across all government service and data sources.

In line with the Manitoba model, a first step forward would be to facilitate linkages between key indicators via the use of a unique identifier. Data collected during pregnancy, at age 1½–2 years (vaccination), at school entry (age 6 years), at the end of elementary school, and at the end of high school could be matched in a secure space. The repository should rely on (a) routinely collected indicators (e.g., birth records, standardized academic tests in elementary school, government high school leaving exams); and (b) brief assessments of normative development in three spheres (physical, cognitive/academic, socio-emotional).

## A stepped intervention strategy

As presented in Tremblay’s paper (Tremblay, 2021), much can be done in terms of preventive intervention from conception onwards. Many are the lost opportunities for gentle, non-invasive, non-stigmatizing services that could modify developmental trajectories in high-risk children. The availability of reliable data on indicators of children’s learning losses, well-being, and health is the critical first step in implementing interventions tailored to the needs of distinct groups.

The data needed to monitor indicators of learning and well-being are shown in Fig. 1. With this information, community-level intervention can be envisioned. A stepped intervention approach proposes that the most effective yet least resource-intensive intervention is delivered first, only “stepping up” to more intensive and targeted intervention as required. The rectangles at the bottom, from outer to inner, represent Level 1 interventions (universal), Level 2 interventions (preventive for individuals with personal risks and health promotive for individuals with social risks), and Level 3 interventions (therapeutic/specialized care).

**Fig. 1:**
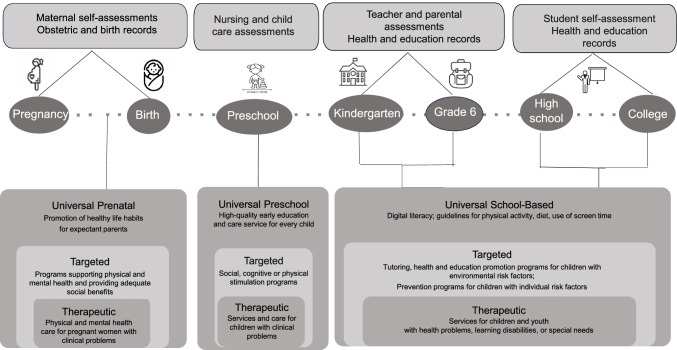
Monitoring and intervention strategies in child education and health in the post-pandemic recovery period: from universal to targeted strategies

We revisited the concept of graded interventions with the objective of mitigating the impact of the pandemic on child development. The four levels of intervention shown in Table 1 present a gradient in the intensity of the intervention and the size of the population targeted: from universal where the entire population is exposed (e.g., childcare services), to targeted groups, to individuals in need.

**Table 1:** Examples of programs and services in the four levels of intervention by the type of risk and population targeted

	Level of intervention	Aim	Type of risk targeted	Individuals targeted	Settings	Examples of interventions by developmental period		
						*Prenatal: Pregnancy*	*Preschool: Early childhood*	*School: Childhood, adolescence*
1	Universal: entire population of families and stakeholders	Health promotion interventions: Increasing exposure to protective factors	None	All, from conception to the end of adolescence	Public spaces, clinics, child care services, schools	Promote/nudge healthy behaviours during pregnancy	Establishment of structured and high-quality early education settings	Improve the quality of education and care
2	Targeted: socio and economic risks	Health promotion interventions to foster empowerment and reduce socio-economic risks	Environmental	Living in socio-economically disadvantaged environments	Low-income neighbourhoods: public health and community settings	Increase social benefits, empower populations living in disadvantaged areas	Facilitating use of child care in socio-economically disadvantaged families	School-level programs to boost the quality of the educational environment
3	Targeted: individual risks	Preventive interventions: Reduce the severity of individual risk factor	Individual	Children or parents with personal risks (e.g., subclinical physical or socio-emotional problems, learning difficulties	Public health and doctors’ clinics, child care services, schools	Prevention program for expecting parents with individual risks (e.g., preventing post-partum depression)	Social skills interventions for children at risk of behavioural problems	Tutoring for children with learning delays/challenges
4	Targeted: individuals with clinically severe problems	Therapeutic interventions: Reduce the severity or eliminate a clinical problem	Individual	Children with identified learning, mental health or health problems	Specialized services in clinics and schools	Treatment of mental and physical health problems in pregnant women	Psychoeducation interventions in child care for children with developmental issues/special needs	Remedial education/special education

### Level 1: Universal health promotion interventions

The first level of intervention applies to the entire population of families in a given territory, regardless of environmental or individual risk factors. Examples include access to high-quality child care and education services prior to school entry for every child, promotion of digital literacy and outdoor education, and nudging strategies to encourage age-appropriate guidelines for intensity and frequency of physical activities and use of electronic devices (Canadian Society for Exercise Physiology, 2021). The post-pandemic recovery is an opportunity to reinforce and extend basic Level 1 interventions, at a time when parents, educators, and professionals yearn for extra guidance.

### Levels 2 and 3: Targeted preventive intervention: environmental and individual risk

The second level of intervention concerns environmental risks, such as living in a socio-economically deprived neighbourhood. The third applies to personal risk factors, such as learning or socio-emotional challenges. These two risk levels may at times overlap, but not always. For instance, most socio-economically disadvantaged children do well, and children with developmental issues are not necessarily from socio-economically disadvantaged families. However, the odds of mental/physical and/or academic challenges are higher in disadvantaged populations as compared with non-disadvantaged (Laurin et al., 2015; Orri et al., 2019). Interventions should include health promotion programs to empower individuals exposed to risks and boost the quality of an otherwise deprived environment, while preventive action works to further develop personal skills.

Tutoring programs have shown high efficacy in addressing learning loss and are being implemented across the world. Evidence from populations most affected by school disruptions suggests that tutoring be offered to children/teens in socio-economically disadvantaged environments (Level 2) or to children/teens with prior learning difficulties (Level 3). However, simply implementing a tutoring program is insufficient. Meta-analyses and systematic reviews have identified various conditions for significant results (Cheung et al., 2021; Gersten et al., 2020; Pellegrini et al., 2021). As pointed out by Haeck and Larose (2021), tutoring by teachers and education professionals during school hours has greater impact than tutoring by peers or adult volunteers (Cheung et al., 2021; Gersten et al., 2020; Pellegrini et al., 2021). Given the lack of teachers in most school boards, however, individuals without formal training are being called upon. Evidence suggests that providing adequate training and supervision to non-professionals can be helpful (Nickow et al., 2020)*.* As regards mental health and well-being, studies have shown promising results and feasibility for cognitive behavioural approaches, including virtual mindfulness interventions (Malboeuf-Hurtubise et al., 2021a, b).

### Level 4: Targeted therapeutic intervention

The fourth level applies to individuals with clinically severe problems that require specialized and intensive educational or health interventions. Level 1–3 interventions can nonetheless reduce the number of individuals affected; in particular, by offering adequate care to expectant mothers with physical (e.g., diabetes) or mental health problems (e.g., clinical depression, anxiety, substance abuse). Nevertheless, there will always be a need for individual high-quality therapeutic services, both in the clinic (pediatric care, physio/occupational therapy) and in school (psychoeducation, remedial tutoring).

Chadi et al. (2021) insist on a pressing need for government investment to support, improve, and adapt existing mental health treatments for an efficient response to the demand created by COVID-19 lockdowns, including a shift towards digital health therapies. Monitoring of the long-term effectiveness of in-person and online services for youth mental health will be key to understanding whether virtual therapeutic solutions should be scaled up.

## Conclusion

Available data suggest that the impact of the COVID-19 pandemic on learning, health, and well-being in children and teens varies according to domain (e.g., mental health vs. learning loss) and individual risk (e.g., socio-economic status vs. personal factors). To date, data on learning loss indicates that children/teens from socio-economically disadvantaged families and/or with prior learning disabilities have suffered the most. As we move forward, we need an intersectoral monitoring system that will distinguish transient from long-term consequences of pandemic-related lockdown measures. Second, we should be ready to deploy a stepped strategy to intervention, ranging from universal promotive interventions to more intensive and targeted interventions, and to specialized remedial or therapeutic care. In particular, as recommended by the United Nations (United Nations, 2021b) and the Lancet Task Force on COVID-19 (Aknin et al., 2021), expectant parents should be offered spaces in high-quality early childcare and education services to (a) maximize parent and child personal and professional growth; and (b) ensure adequate access to preschool preventive services. Third, children living in socio-economically deprived neighbourhoods or those with individual risk factors (mental health challenges, learning delays) should be offered programs promoting mental and physical health. In particular, academic tutoring should be easily accessible in socio-economically disadvantaged settings. Finally, medical care and educational remediation services should be adapted to accommodate the potentially higher number of children with clinically severe problems. Importantly, as expectant parents and children/teens with mental/physical health risks are disproportionally found in low socio-economic contexts, the interventions and services modifying lived environments are key to reducing social inequalities in present and future generations.

## Comprendre et atténuer les répercussions de la pandémie de COVID-19 sur les enfants : un plan pour réduire les inégalités du développement

Le Secrétaire général des Nations Unies a qualifié l’impact des interruptions scolaires en raison de la pandémie de la COVID-19 de « catastrophe générationnelle » (United Nations, 2021a). Les enfants, les adolescents et les jeunes adultes seront-ils en mesure de se rattraper facilement, sans intervention particulière? Ou risquent-ils de subir des conséquences sérieuses à long terme, nécessitant un soutien intensif? Les attentes habituelles de la société à l’égard de la jeunesse devront-elles être réduites? La *Section spéciale sur un plan de relance pour les enfants suite à la pandémie* présente quatre articles sur les conséquences de la pandémie. Ce commentaire résume les préoccupations principales des articles et propose un système de surveillance pour mieux comprendre les impacts à court terme et à long terme, ainsi qu’une série d’interventions pour les atténuer.

Les auteurs des quatre articles joignent leur voix à celles des nombreux organismes nationaux et internationaux, dont les Nations unies, qui soulignent l’importance de l’accès aux données permettant de suivre de près le développement, l’éducation et le bien-être des enfants et des jeunes au cours de la prochaine décennie. Les dernières données internationales tendent à confirmer ce que nombreuses personnes présumaient – un impact disproportionné de la pandémie sur les enfants les plus vulnérables. Toutefois, l’ampleur de l’impact varie en fonction du type de vulnérabilité individuelle, du domaine de fonctionnement étudié, et la durée du confinement. Afin de bien saisir l’ampleur de l’impact, il nous faudra combler des lacunes d’information dans des domaines clés.

## Définir les priorités : qui et quoi cibler pour les efforts de relance

Presque tous les enfants et adolescents auront vécu des perturbations majeures en ce qui concerne leur scolarisation, leurs activités sociales, et leurs activités physiques.Toutes les sphères écologiques du fonctionnement humain auront été touchées. Toutefois, les conséquences mises en lumière à ce jour par les données populationnelles sont nuancées et rapportent des impacts plus marqués dans le domaine de l’apprentissage que dans celui de la santé mentale.

### Question 1 : Retards d’apprentissage

En date du 29 mars 2021, les écoles des différentes provinces au Canada avaient fermé leurs portes pour des durées allant de 40 à 100 jours à temps plein; ceci sans compter la modalité de jours de classes alternés au secondaire et les fermetures de classe en raison d’éclosions (Gallagher-Mackay et al., 2021). L’enseignement à distance fut déployé de manière inégale, et n’a engagé que 50 % des étudiants ou moins, comparé à 80 % des étudiants pendant l’école en présentiel (Naitre et Grandir, 2021). Haeck et Larose (Haeck & Larose, 2021) s’attendent à des pertes d’apprentissage considérables chez tous les enfants/adolescents, avec un accroissement des inégalités de réussite de 30 % entre les élèves selon leurs difficultés académiques. Les rares études de bonne qualité mettent en évidence une baisse des notes scolaires allant de 2 à 6 percentiles pour l’ensemble des enfants et de 4,5 à 17 percentiles pour les enfants issus de familles dont les parents ont un faible niveau d’éducation ou parmi ceux fréquentant des écoles issues de milieux défavorisés (Engzell et al., 2021; Lewis et al., 2021).

En outre, certaines données suggèrent que les normes pour l’obtention d’un diplôme ont été ajustées pour correspondre au niveau de la population. Par exemple, les taux de graduation du secondaire dans différents pays ont été aussi élevés, sinon plus, en 2020 qu’au cours des années précédentes (UNESCO et al., 2020). De plus, les curriculums scolaires ont été ajustés pour cibler les apprentissages essentiels. Ainsi, les conséquences de la pandémie ne se feront ressentir qu’en comparant la situation aux épreuves standardisées pré- et post-pandémie. Ces évaluations serviront à déceler les effets à moyen terme des interruptions scolaires et à surveiller les progrès des élèves au cours de la relance, particulièrement en ce qui concerne les élèves les plus vulnérables.

### Question 2 : Bien-être et santé mentale

La deuxième question qui se pose concerne l’impact de la pandémie sur la santé mentale et le bien-être des enfants. Une méta-analyse de 136 études menées au cours de la première année de la pandémie (entre mars 2020 et février 2021) suggère que les taux de dépression et d’anxiété cliniquement élevés chez les jeunes (<18 ans) ont doublé par rapport aux niveaux prépandémiques. Un jeune sur quatre dans le monde présenterait des symptômes de dépression, tandis qu’un jeune sur cinq présenterait des symptômes d’anxiété. Ces estimations, qui ont augmenté au fil du temps, sont plus élevées pour les enfants plus âgés et pour les filles (Racine et al., 2021). Par ailleurs, les données longitudinales indiquent qu’en population générale, les groupes les plus impactés ne sont pas nécessairement ceux qui présentaient les plus graves problèmes de santé mentale avant la pandémie (Watkins-Martin et al., 2021). Par exemple, 48 % des parents signalent des « défis » de santé mentale de novo chez leurs enfants (Inspiring Healthy Futures, 2021). Notez que cette dernière étude porte sur les inquiétudes des parents et non pas sur des évaluations cliniques.

Dans le même ordre d’idées, la consommation de substances n’a pas nécessairement évolué de façon uniforme; la trajectoire dépend plutôt du type de substance et du mode de consommation. En particulier, l’abus d’alcool chez les jeunes adultes semble avoir diminué (Pocuca et al., under review), probablement en raison de la diminution des opportunités sociales due au confinement. Les données sur le suicide varient également selon différents paramètres, tels que le type de données et leur provenance. Chadi et al. (2021) décrivent une augmentation des tentatives de suicide (données hospitalières) mais pas d’augmentations des taux de décès par suicide. Des recherches à plus long terme seront nécessaires afin de clarifier pour qui les effets sur la santé mentale sont de longue durée.

### Question 3 : Saines habitudes de vie

Le milieu scolaire comporte des normes et des rythmes de vie qui soutiennent les interactions sociales et les comportements sains (entre autres, l’alimentation et l’activité physique), à la fois pendant les heures de classe et pendant les activités parascolaires. Selon Gauvin et al. (2021), les fermetures associées à la pandémie ont considérablement réduit l’activité physique, et augmenté les comportements sédentaires, le temps d’écran, la consommation de friandises et la malbouffe. Les réductions de l’activité physique semblent plus prononcées parmi les enfants de milieux défavorisés, surtout dans les zones urbaines (Gauvin et al., 2021). Il n’est donc pas surprenant qu’une étude américaine récente reposant sur un large échantillon (*n* = 191 509) d’enfants de 5 à 15 ans montre une augmentation moyenne de l’indice de masse corporelle de 2,3 kg (de plus qu’en période pré-pandémique) ainsi qu’une augmentation de 8,7 % de l’obésité (Woolford et al., 2021).

On constate une augmentation du temps d’écran depuis le début de la pandémie (Sultana et al., 2021), en outre en raison du travail en classe qui est souvent en ligne. Toutefois, le problème du ‘temps écrans’ se pose surtout pour les activités de loisir. En éducation, il est essentiel de prioriser la littératie numérique (OECD, 2021) : c.-à-d. l’intérêt, l’attitude, et la capacité d’un individu à utiliser la technologie numérique afin d’accéder de façon appropriée aux informations, de construire de nouvelles connaissances et de communiquer avec les autres (Government of British Columbia, 2021). Chez les enfants, la littératie numérique doit faire partie des curriculums éducatifs, alors que l’utilisation des appareils électroniques à des fins ludiques doit être encadrée et surveillée par des adultes.

## Stratégie de surveillance harmonisée

Tel qu’indiqué dans le plan de relance post-pandémie proposé par l’ONU, l’investissement dans les systèmes et les infrastructures permettant l’analyse de données populationnelles sera le pivot des efforts de reprise (United Nations, 2020). Tous les auteurs de cette section spéciale soulignent l’importance de la recherche pour différencier les effets transitoires des effets persistants de la pandémie.

Des travaux devront être entrepris afin de combler les lacunes et de surmonter les défis suivants : 1) la difficulté d’utilisation des données administratives, en particulier pour les études intersectorielles (p. ex. santé et éducation); et 2) le manque de données longitudinales. À priori, les données clés pour étudier le développement des enfants se trouvent dans différentes bases de données qui sont générées et gérées par différents groupes et organismes (chercheurs, services de santé et psychosociaux, écoles, ministères). Le premier défi se présente lorsque l’on veut étudier des questions intersectorielles telles que l’efficacité d’une mesure éducative sur la santé. Les données médicales et scolaires dont on a besoin se retrouvent dans différents systèmes et peuvent difficilement être appariées. Le deuxième défi (données longitudinales) se présente à chaque fois qu’on essaie de déterminer la trajectoire des soins ou de l’éducation au cours du développement de l’enfant. Par exemple : afin d’évaluer un changement de politiques concernant l’éducation au cours de la petite enfance – à savoir si le changement est associé à une meilleure préparation à l’entrée scolaire, à des taux de diplomation plus élevés, à une moins forte utilisation des services psychosociaux – il faut avoir accès aux données sur l’utilisation des services à la petite enfance pendant les cinq premières années de vie, ainsi qu’aux données administratives en santé et en éducation pendant l’école primaire et secondaire. Un système de surveillance harmonisé permettrait de suivre les indicateurs de l’apprentissage et du bien-être de façon longitudinale.

Dans la plupart des territoires/provinces, l’absence d’un système de surveillance longitudinale harmonisé pour le développement des enfants est un obstacle majeur à l’implantation des services préventifs et/ou thérapeutiques précoces. Au Québec, l’Institut de la Statistique du Québec porte le mandat de faciliter l’accès aux données administratives et l’appariement intersectoriel des données. Les travaux en ce sens vont bon train. Par ailleurs, le meilleur exemple au Canada est le *Manitoba Population Research Data Repository* (University of Manitoba, 2020), une plateforme regroupant des données administratives en santé et en éducation, les états civils, et les enquêtes concernant les résidents de la province. Un identifiant unique – le *Personal Health Identification Number* (PHIN) – facilite le jumelage des données de toute provenance gouvernementale et autres sources, de la naissance à l’âge adulte.

Conformément au modèle manitobain, l’utilisation d’un identifiant unique, ainsi que d’un environnement sécurisé, s’avère critique dans la mise en place d’un système permettant l’utilisation intersectorielle des données. Un tel système pan-canadien permettrait l’arrimage des données saisies au cours de la grossesse, à la petite enfance (p. ex. 18–24 mois lors de la vaccination), à l’entrée à l’école (âge 6 ans), à la fin de l’école primaire, à la fin du secondaire et à l’âge adulte. La stratégie de surveillance devrait s’appuyer sur : a) des informations collectées de manière routinière (par exemple, registres de naissance, épreuves uniformes à l’école élémentaire et au secondaire); et b) de brèves évaluations du développement normatif dans trois domaines (physique, cognitif/académique, socio-émotionnel).

## Une stratégie d’intervention par niveau

Tel que présenté dans l’article de Tremblay (Tremblay, 2021), de nombreuses interventions préventives, c.-à-d. des services non invasifs et non stigmatisants, pourraient être mis en place dès la grossesse. L’obtention de données fiables pour indiquer les pertes d’apprentissage, le bien-être, et la santé constitue un premier pas essentiel à l’implantation d’interventions adaptées aux groupes ayant des besoins distincts.

Muni de l’information tirée des indicateurs d’apprentissage et du bien-être, le modèle de la Fig. 1 propose, en premier lieu, des interventions s’adressant à toute une population. L’approche interventionnelle par niveau propose que l’intervention à la fois la plus efficace et exigeant le moins de ressources soit appliquée en premier lieu. Ensuite, au besoin, d’autres strates d’interventions progressivement plus intensives et plus ciblées pourraient être mises en œuvre. Les rectangles au bas de la Fig. 1, en partant de l’extérieur vers l’intérieur, représentent les interventions de Niveau 1 (universelles), Niveau 2 (ciblées) et Niveau 3 (thérapeutiques).

**Fig. 1:**
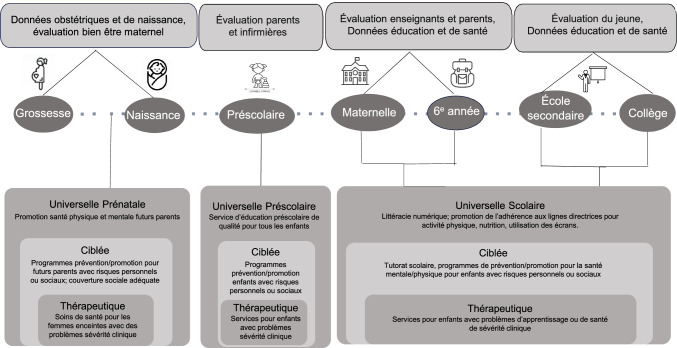
Stratégies de surveillance et d’interventions préventives par niveau pour enfants/adolescents pendant la relance post-pandémie : de l’universel au ciblé

Le tableau 2 présente le concept de l’intervention par niveau tel qu’appliqué à l’atténuation de l’impact de la pandémie sur le développement de l’enfant. Les quatre niveaux d’intervention représentent un gradient dans l’intensité de l’intervention et dans la taille de la population ciblée, passant de l’universel où la population entière est exposée (ex. éducation préscolaire), aux groupes ciblés, à l’individu ayant des besoins particuliers.

**Table 2:** Quatre niveaux d’intervention ciblant différentes populations et différents facteurs de risque

Niveau d’intervention		Objectif	Type de risque ciblé	Population ciblée	Cadres d’intervention	Exemples d’interventions par stade développemental		
						*Prénatal : Grossesse*	*Préscolaire : Petite enfance*	*Scolaire : Enfance et adolescence*
1	Universel : population entière de familles et de parties prenantes	Interventions de promotion de la santé : augmenter l’exposition aux facteurs protecteurs	Aucun	Population entière, de la conception à la fin de l’adolescence	Espaces publics, cliniques, CPE, écoles	Promotion des comportements sains pendant la grossesse	Mise en place de CPE structurés de qualité	Amélioration de la qualité de l’éducation et des soins
2	Ciblé : risques socioéconomiques	Interventions de promotion de la santé : favoriser l’autonomisation et réduire les risques socioéconomiques	Environnemental	Milieux socioéconomiques défavorisés et/ou marginalisés	Quartiers défavorisés : cliniques de santé publique, centres communautaires	Augmentation des bénéfices sociaux; habiliter les populations des quartiers défavorisés	Mesures spéciales facilitant l’utilisation des services de garde pour les familles de milieux défavorisés	Programmes scolaires pour stimuler la qualité de l’éducation et de l’environnement
3	Ciblé : risques individuels	Interventions préventives : réduire la sévérité des facteurs de risque individuels	Individuel	Enfants ou parents avec risques individuels (ex. problèmes sous clinique)	Cliniques de santé publique, cliniques médicales, CPE, écoles	Programme de prévention pour futurs parents avec risques individuels (ex. prévention de la dépression post-partum)	Interventions de compétences sociales pour les enfants à risque de troubles du comportement	Tutorat pour enfants avec retards d’apprentissage / défis
4	Ciblé : individus avec troubles cliniques sévères	Interventions thérapeutiques : réduire la sévérité ou éliminer certains troubles cliniques	Individuel	Enfants avec troubles d’apprentissage, problèmes de santé mentale ou physique	Services spécialisés en clinique ou en milieu scolaire	Traitement des troubles de santé mentale et physique chez la femme enceinte	Interventions psychoéducation services de garde pour les enfants avec troubles du développement	Remédiation/éducation spécialisée

### Niveau 1 : Interventions universelles de promotion de la santé

Le premier niveau d’intervention s’applique à l’ensemble de la population; c.-à-d. à toutes les familles dans un territoire donné, quel que soit le risque environnemental ou personnel. Voir par exemple, l’accès pour tout enfant à l’éducation préscolaire de qualité; la promotion de la littératie numérique et de l’éducation en plein air; ainsi que les stratégies d’encouragement pour l’adhérence aux lignes directrices appropriées à l’âge sur l’intensité et la fréquence de l’activité physique et l’utilisation des appareils électroniques (Canadian Society for Exercise Physiology, 2021). La relance post-pandémie constitue l’opportunité idéale pour mettre en place et renforcer les interventions de Niveau 1, à l’heure où les parents, éducateurs, et professionnels sont à la recherche de conseils pratico-pratiques.

### Niveaux 2 et 3 : Interventions préventives ciblées – environnement et risque personnel

Le deuxième niveau d’intervention s’applique aux risques environnementaux sur le plan socioéconomique, tel que l’habitation dans un milieu défavorisé. Le troisième niveau s’applique aux facteurs de risque individuel, tel que vivre avec des défis d’apprentissage ou socioémotifs. Les deux vont souvent de pair, mais pas toujours. La plupart des enfants de milieux défavorisés réussissent à l’école et sur le marché du travail, et les enfants atteints de troubles du développement viennent de tous les milieux. Cependant, la probabilité de problèmes de santé mentale ou physique et/ou de difficultés d’apprentissage est plus élevée parmi les populations défavorisées (Laurin et al., 2015; Orri et al., 2019). Les interventions devraient inclure des programmes de promotion de la santé pour modifier les environnements défavorisés; et de prévention pour développer les compétences individuelles.

Les programmes de tutorat se sont montrés efficaces pour combler les retards scolaires et sont actuellement implantés largement au Canada et à l’international. Les données sur les populations les plus affectées par les perturbations scolaires suggèrent que le tutorat aux enfants/adolescents de milieux défavorisés sur le plan socioéconomique (Niveau 2) et aux enfants/adolescents avec des antécédents de difficultés d’apprentissage (Niveau 3) est une stratégie efficace, avec des effets de taille moyenne (d de cohen = 0.3, Cheung et al., 2021; Gersten et al., 2020; Pellegrini et al., 2021). Les études indiquent que certaines conditions doivent toutefois être respectées (voir Cheung et al., 2021; Gersten et al., 2020; Haeck & Larose, 2021; Pellegrini et al., 2021), incluant le fait que ce soit les enseignants qui offrent le tutorat (dans la mesure du possible), ou encore que le personnel non enseignants (tels que des étudiants) soit bien formé et encadré (Nickow et al., 2020). Enfin, en ce qui concerne la santé mentale et le bien-être, les études indiquent des résultats prometteurs pour les approches cognitivo-comportementales, y compris les interventions virtuelles de pleine conscience (Malboeuf-Hurtubise et al., 2021a, b).

### Niveau 4 : Interventions thérapeutiques ciblées

Le quatrième niveau d’intervention s’applique aux individus avec des troubles de sévérité clinique qui requièrent des interventions spécialisées. Notons que les interventions de Niveau 1 à 3 ont la capacité de réduire le nombre d’individus qui présenteront des difficultés cliniques. À titre d’exemple, les soins adéquats pour les femmes enceintes pourraient réduire le diabète gestationnel et les problèmes de santé mentale maternelle (dépression clinique, anxiété, abus de substances) ainsi que les conséquences sur l’individu, la famille, et l’enfant à naitre. Ceci dit, il y aura toujours un besoin de services thérapeutiques individuels de qualité à la fois en clinique (soins pédiatriques, physio- et ergothérapies) et en milieu scolaire (psychoéducation, orthopédagogie).

Chadi et al. (2021) insistent sur le besoin pressant d’investissements gouvernementaux pour soutenir, améliorer, et adapter les traitements de santé mentale existants pour une réponse efficace à la demande créée par les perturbations pandémiques de la COVID-19, y compris la téléthérapie. Le suivi de l’efficacité à long terme des services en présentiel et en ligne pour la santé mentale des jeunes sera la clé pour savoir si oui ou non il faut élargir les services de thérapie virtuelle.

## Conclusion

Les données à ce jour suggèrent que l’impact de la pandémie COVID-19 sur l’apprentissage, la santé, et le bien-être des enfants et des adolescents variera selon le domaine du développement (ex. santé mentale c. apprentissage) et selon le type de facteur de risque (ex. risques socioéconomiques c. personnels). Les études récentes montrent que les enfants/adolescents avec des antécédents de troubles d’apprentissages et/ou de familles défavorisées sur le plan socioéconomique en souffriront le plus. Trois lignes d’action prioritaires émergent à la lumière des connaissances scientifiques actuelles. Premièrement, on souligne le besoin d’accéder à données intersectorielles permettant de distinguer les conséquences à court terme c. à long terme des perturbations pandémiques. Deuxièmement, il nous faut être prêts à déployer une stratégie interventionnelle par niveau, avec des interventions universelles en promotion, jusqu’aux interventions plus ciblées et intensives. En particulier, et tel que recommandé par les Nations Unies dans le cadre des objectifs de développement durable (United Nations, 2021b), ainsi que par le groupe de travail du Lancet sur la COVID (Aknin et al., 2021), la disponibilité d’un réseau structuré d’éducation préscolaire est essentielle à la réduction des inégalités sociales et à la relance pour l’éducation et la santé. Troisièmement, il faudra offrir aux enfants/adolescents, nottamment à ceux de milieux défavorisés ayant des facteurs de risque personnels (défis de santé mentale, retards d’apprentissage) des programmes favorisant la santé mentale et les saines habitudes de vie. En particulier, le tutorat scolaire devrait être facilement accessible dans tous les milieux défavorisés. Enfin, les services de soins médicaux et de remédiation éducative devraient être adaptés pour accommoder le nombre potentiellement plus important d’enfants/ados avec des troubles cliniques sévères.
